# American Institute of Homœopathy

**Published:** 1899-01

**Authors:** Benj. L. Bailey, Eugene H. Porter

**Affiliations:** President; Secretary


### AMERICAN INSTITUTE OF HOMŒOPATHY.

JANUARY 1ST, 1898.

The coming session of the American Institute of Homœopathy will be the fifty-fifth session in its history. Organized with scarcely a hundred of our fellows, to foster and spread the tenets of our school, it finds itself to-day the organized body of nearly or quite 20,000 acknowledged practitioners of the homœopathic faith. Yes, the entire body of the profession has been called upon the stage since the organization of this, our grand old institute, the oldest national medical society in the United States. What has it done for us? It has inspired noble fathers with a courage, a faith, a conviction, and has given to us a heritage, a knowledge, a conception of the greatest law of cure, and a most honorable place in the world as homœopathic physicians. It has raised the standard of medical education; it has molded just and kindly legislation; it has swept away the barriers and opened to us every honorable place that awaits an honorable profession; it has given us a literature; it has made us what we are. And what have we, its children, done for the American Institute of Homœopathy? In its fifty-five years, perhaps 4,000 of the many thousands who in all these years have avowed allegiance to our master, have for a greater or lesser time been members of the Institute. But only the few have been faithful laborers over many years. The greater number have reaped where others have sown. We cannot believe it is aught but the carelessness and neglect of busy life, but had not the Institute molded public opinion, corrected legislation, and builded for education, how many of us would have had the opportunities for a busy, prosperous life such as we have led? We ask you, who though brothers, are not members with us, to give us your support, and to render unto the American Institute, which has cherished you and your interests, that which is its due. From you, fellow-members, we ask special and personal work. We ask you in every city of the land to arrange to meet your fellows in social gatherings or around the banquet board on the evening of Wednesday, January 25th, 1899. Let the evening be given to the recalling of the past work of the American Institute, to plans and vows of loyalty for the future, to a seeking of new members, to a recognition of the strength of a united force, to the giving up of the selfishness and thoughtlessness of the individual, to the cultivation of a labor not for ourselves, but also

"for others." The knowledge that on this one evening throughout the breadth of our land we are all giving ourselves to a common cause, may give to Homœopathy and to the American Institute an impetus that shall enable her to place the child of her love and care on a foundation as firm and strong as the granite hills. And may the medical press of February, 1899, give us reports of hundreds of meetings full of enthusiasm and loyalty, that shall sound from ocean to ocean.

BENJ. L. BAILEY, *President.*

EUGENE H. PORTER, *Secretary.*

